# Supplementary material for: Prenatal care providers’ perceptions of the SARS-Cov-2 vaccine for themselves and for pregnant women
Source: PLoS One. 2021 Sep 13;16(9):e0256080. doi: 10.1371/journal.pone.0256080 (PMC8437278; doi:10.1371/journal.pone.0256080)
Supplement: S1 Fig — (DOCX) [file pone.0256080.s001.docx]

**SURVEY**

*Translated from the French*

Profession:

Obstetrician and Gynaecologist

General practitioner

Midwife

Age

Gender:

Male

Female

I do not want to specify

Year of Graduation

Place of activity:

University hospital

General hospital

Private clinic

Private practice

Mixed private / public

Number of your exercise department

Usually, do you get the flu shot?

YES

NO

Do you usually prescribe the flu shot to pregnant women?

YES

NO

As a healthcare worker, would you agree to be vaccinated against SARS-CoV-2?

Yes

Yes because I am a person considered at risk

No because I don't have risk factors

No

If not, for what reasons would you not agree to be vaccinated against the SARS-CoV-2? (multiple choice is possible)

More afraid of the side effects of the vaccine than the disease

Not enough inputs on side effects

No sufficient inputs on efficiency

waiting information from professional societies

Waiting for other sources of information

In the current state of knowledge, would you prescribe vaccine against SARS-CoV-2 to pregnant women?

Yes for all pregnant women seen in consultation

Yes Only for pregnant women with Risk factors ((age> 35, obesity, arterial hypertension, diabetes, obesity)

No

If not why would you not prescribe the vaccine against SARS-CoV -2 to pregnant women?

I am waiting the opinion of professional societies

I am waiting ministerial recommendations

Because it is not a population at risk of severe form

Due to the type of vaccine available to date

No for other reasons

More afraid of the side effects of the vaccine than the disease

Not enough inputs on side effects

No sufficient inputs on efficiency

Fear of a teratogenic effect of the vaccine

Vaccination is currently not recommended by the “Haute Autorite de Sante” lth in this population

Not informed enough

Would you prescribe the vaccine against SARS-CoV -2t to women willing to become pregnant?

Yes for all women seen in consultation

Yes but only for women with risk factors

No

If all of these vaccines were available and you had a choice, which one would you prescribe during pregnancy?

BioNTech-Pfizer yes / no

Moderna mRAN-1273 yes / no

AstraZeneca and Oxford AZD1222 yes / no

J and J-Janssen Ad23.COV2.S yes / no

Novavax yes / no

GSK-Sanofi yes / no
