# Supplementary material for: Prenatal care providers’ perceptions of the SARS-Cov-2 vaccine for themselves and for pregnant women
Source: PLoS One. 2021 Sep 13;16(9):e0256080. doi: 10.1371/journal.pone.0256080 (PMC8437278; doi:10.1371/journal.pone.0256080)
Supplement: Attachment — Submitted filename: RespReviewers.docx [file pone.0256080.s002.docx]

**Response to the editors:**

When submitting your revision, we need you to address these additional requirements.

1. Please ensure that your manuscript meets PLOS ONE's style requirements, including those for file naming. The PLOS ONE style templates can be found at

<https://journals.plos.org/plosone/s/file?id=wjVg/PLOSOne_formatting_sample_main_body.pdf> and

<https://journals.plos.org/plosone/s/file?id=ba62/PLOSOne_formatting_sample_title_authors_affiliations.pdf>

A: We check that the manuscript meets PLOS ONE’s style requirements

2. Please provide additional details regarding participant consent. In the ethics statement in the Methods and online submission information, please ensure that you have specified (1) whether consent was informed and (2) what type you obtained (for instance, written or verbal, and if verbal, how it was documented and witnessed). If your study included minors, state whether you obtained consent from parents or guardians. If the need for consent was waived by the ethics committee, please include this information.

If you are reporting a retrospective study of medical records or archived samples, please ensure that you have discussed whether all data were fully anonymized before you accessed them and/or whether the IRB or ethics committee waived the requirement for informed consent. If patients provided informed written consent to have data from their medical records used in research, please include this information.

A: when answering the questionnaire, the practitioner agreed to participate. We added this sentence in the methods section. The ethical committee agreed to the study.

3. Please state whether you validated the questionnaire prior to testing on study participants. Please provide details regarding the validation group within the methods section.

A: we did not validate the questionnaire which was constructed by the team.

4. In your Data Availability statement, you have not specified where the minimal data set underlying the results described in your manuscript can be found. PLOS defines a study's minimal data set as the underlying data used to reach the conclusions drawn in the manuscript and any additional data required to replicate the reported study findings in their entirety. All PLOS journals require that the minimal data set be made fully available. For more information about our data policy, please see <http://journals.plos.org/plosone/s/data-availability>.

Upon re-submitting your revised manuscript, please upload your study’s minimal underlying data set as either Supporting Information files or to a stable, public repository and include the relevant URLs, DOIs, or accession numbers within your revised cover letter. For a list of acceptable repositories, please see <http://journals.plos.org/plosone/s/data-availability#loc-recommended-repositories>. Any potentially identifying patient information must be fully anonymized.

Important: If there are ethical or legal restrictions to sharing your data publicly, please explain these restrictions in detail. Please see our guidelines for more information on what we consider unacceptable restrictions to publicly sharing data: <http://journals.plos.org/plosone/s/data-availability#loc-unacceptable-data-access-restrictions>. Note that it is not acceptable for the authors to be the sole named individuals responsible for ensuring data access.

We will update your Data Availability statement to reflect the information you provide in your cover letter.

A: The full data are available at:

<https://drive.google.com/file/d/1PdaN4Pxc-8EyNFlvV_9FGexGmerZbpOI/view?usp=sharing>

**Response to the reviewers:**

Reviewer #1: Thank you for the opportunity to review this paper which describes the perceptions of health care providers in relation to COVID-19 vaccination for pregnant women.The study is very timely given the status of the COVID-19 pandemic. Overall I think the study is soundly conducted and worthy of publication. The sample is quite large – 1400 responses including obstetricians, midwives and GPs.

Some terminology requires attention to be clearer. I did not know what ‘collective exercise’ in terms of the medical practice was (this is in Table 1 but also in text). Perhaps this did not translate well. I suggest changing Medical practice to Clinical practice and Collective exercise perhaps to Model of care.

A : Indeed, we did not use the correct terminology. We ask an English-speaking medical doctor from UK who suggest to use the term “group practice.

Reviewer #2: Thank you for the opportunity to review this paper that reports a cross sectional survey exploring the perceptions of French antenatal care providers, regarding the COVID-19 vaccine. The paper is generally well written, the authors are congratulated on this contribution to contemporary and much needed evidence. The following feedback is offered with the view to strengthening the manuscript.
Page 11 “However, to adapt the content of educational pieces before improving knowledge of provider (this is unclear- do you mean to inform education for healthcare professionals?)
… it is recommended to conduct a SURVEY on healthcare workers to understand their views.” Thus, we aimed to INTERVIEW the health care workers (obstetricians, midwives and GPs) involved in the management of pregnant women.

The two data collection methods CAPITALISED contradict each other please amend.

A : we change for “we collect answers of a survey from healthcare workers”
Page 12 Title needs correcting – currently reads as MATERIAL AND METHOS –please include ‘D’ in the word Methods

A: change made

Page 12 - “Haute Autorité de Santé” statement that allowed SARS-CoV-2 for high-risk pregnant women [18]. – do you mean RECOMMENDED COVID-19 vaccination for high- risk pregnant women?

A : we change as suggested.

Page 12 – “risk factor i.e. age up to 35,” – do you mean over 35?

A: yes. Change made

Page 12 – “…obesity, diabetes and essential blood pression” – do you mean blood pressure, or essential hypertension?

Yes. Essential hypertension. Change made.

Page 13 “ their behaviour to the flu vaccination” – do you mean their usual participation in the flu vaccination?

The use of the phrase/ word (collective) exercise throughout the manuscript is unclear- from the context it appears that this might be replaced with the word practice – modality or model of practice/ model of care. A few examples are below – please revise and clarify

Page 13 “ The main modality of exercise was private practice (32.7%), followed by university hospital activity (26.2%) and general hospital activity”
Table 1 is clear – the title ‘collective exercise’ however is not clear - for your consideration- may be better understood as ‘practice setting’, or, is it better as collaborative practice? Suggest English language editorial support
Page 17 “A collective exercise was positively correlated with a positive vaccination
practice among caregivers and their patients and with a positive perception of the SARS-CoV-“ page 18 “ modalities of exercise (collective or individual);” in this instance does collective actually mean collaborative? Does exercise mean practice? Page 21 “ Being an obstetrician in a collective exercise” – this collective exercise does not make sense

A: Indeed, we did not use the correct terminology. We ask an English-speaking medical doctor from UK who suggest to use the term “group practice.

“However, in the context of globalisation and competition between countries, political views can influence vaccine acceptance [32].” – it is not clear what direct relevance this statement has to your study findings – were political views or agents discovered in your findings? If there was no direct link or relevance to your findings, I would suggest removing, otherwise please expand and clarify.

A: We withdrawn this sentence.

Page 23 “In this survey, which was conducted on all employees of a health care system, in December, before…” – is this referring to your study or another one? If it’s about the study in the previous line then maybe clarify … in this US survey.

A: Indeed, It was not clear. We change for “in an US survey”.

Thank you again for your work to produce this great study. I wish you all the best.
